# Temperature Dependence of Faraday Effect-Induced Bias Error in a Fiber Optic Gyroscope

**DOI:** 10.3390/s17092046

**Published:** 2017-09-07

**Authors:** Xuyou Li, Pan Liu, Xingxing Guang, Zhenlong Xu, Lianwu Guan, Guangchun Li

**Affiliations:** 1 College of Automation, Harbin Engineering University, Harbin 150001, China; lixuyou@hrbeu.edu.cn (X.L.); guangxingxing@hrbeu.edu.cn (X.G.); guanlianwu@hrbeu.edu.cn (L.G.); lgc_67@hrbeu.edu.cn(G.L.); 2 Shandong Institute of Space Electronic Technology, Yantai 264000, China; xzlsdlg@163.com

**Keywords:** fiber optics sensors, gyroscope, Faraday effect, magnetic fields, bias errors

## Abstract

Improving the performance of interferometric fiber optic gyroscope (IFOG) in harsh environments, such as magnetic field and temperature field variation, is necessary for its practical applications. This paper presents an investigation of Faraday effect-induced bias error of IFOG under varying temperature. Jones matrix method is utilized to formulize the temperature dependence of Faraday effect-induced bias error. Theoretical results show that the Faraday effect-induced bias error changes with the temperature in the non-skeleton polarization maintaining (PM) fiber coil. This phenomenon is caused by the temperature dependence of linear birefringence and Verdet constant of PM fiber. Particularly, Faraday effect-induced bias errors of two polarizations always have opposite signs that can be compensated optically regardless of the changes of the temperature. Two experiments with a 1000 m non-skeleton PM fiber coil are performed, and the experimental results support these theoretical predictions. This study is promising for improving the bias stability of IFOG.

## 1. Introduction

Inertial sensors for rotation are used widely in commercial and military systems. In the tactical applications market, the microelectromechanical systems (MEMS) gyroscope [1,2,3] and resonant micro optical gyroscope (RMOG) [4,5,6] have attracted much attention due to compactness, light weight, and low-cost. In the high-performance market, the ring laser gyroscope (RLG) and the interferometric fiber optic gyroscope (IFOG)—which have advantages of broad dynamic range of velocity measurements, insensitivity to accelerations and overloads, short warm-up time, etc.—are superior to the traditional mechanical gyroscope [7]. However, the RLG poses challenges in that they require specialized components, such as superb mirrors, helium-impermeable low expansion glass, and high-voltage discharge electrodes [8,9,10]. As opposed to RLG, the IFOG is a truly solid-state device using semiconductor light sources as opposed to high voltage gas lasers, and therefore offers greater reliability. The IFOG is used in a variety of applications, including ship and sub-sea inertial navigation, along with stabilization and positioning, and it is expected to become the ultimate rotation sensing technology [11,12]. 

Despite these achievements, the adaptability of IFOG in the harsh environment (thermal perturbations, vibration transients, and the Earth’s varying magnetic field) can still be improved further to broaden its application market, including navigation of aircraft [13,14,15]. Under the action of magnetic field, nonreciprocal phase shift may occur through the Faraday effect in the fiber. Because the magnetic field of the fiber coil change with time and space, the Faraday effect results in an error in the IFOG output signal that is indistinguishable from the angular rate. In 1986, Hotate et al. reported that the Faraday effect-induce bias error is depended to the random twist of the birefringence of the polarization-maintaining (PM) fiber [16]. Unfortunately, the catch is inevitably in the process of drawing the fiber and winding the fiber coil. Although the Faraday effect is weak in silica, it is strong enough to be troublesome in the high-sensitivity IFOG [17]. For a polarization maintaining IFOG (PM-IFOG), the typical magnetic sensitivity is on the order of 10°/h/mT, well above the navigation-grade requirement of 0.1°/h/mT [18,19]. 

Generally, to minimize the Faraday effect-induced bias error, some methods can be adopted. The first method is to place the fiber coil in the *μ*-metal magnetic shield [20]. The state-of-the-art technology is to use multiple layers of magnetic shield structure with the cost of increasing both weight and size of the IFOG. The second method is to replace the solid-core, index-guided fiber by the air-core photonic bandgap fiber (PBF) [21,22]. Because most of the fundamental mode is confined in air, the PBF has a much lower Verdet constant than silica. The third method is to use magnetic compensation coils, which present particular magnetic sensitivity magnitude and direction [23]. When the compensation coils are positioned properly with respect to the inherent sensing coil of IFOG, the magnetic sensitivity of IFOG can be eliminated. The fourth method is the optical compensation approach [24,25]. The Faraday effect-induced bias errors of two polarizations in PM fiber possess opposite polarities, thus they can be canceled out optically when both polarizations are utilized simultaneously.

In practical application, the effect of the thermal perturbations and the magnetic field coexist in IFOG. Recently, nonreciprocal phase error caused by magnetic-thermal coupling has been reported [26]. Under the combined action of magnetic-thermal physical fields, the thermal stress-induced birefringence changes with the variation of temperature due to the different expansion between fiber coil skeleton and fiber material. Thus, the Faraday effect-induced bias error of PM-IFOG changes with temperature variation in fiber coil with skeleton. Actually, in order to suppress the thermal stress introduced by the fiber coil skeleton, the skeleton can be taken off in the process of manufacturing the fiber coil. To the best of our knowledge, the temperature dependence of the Faraday effect-induced bias error in the non-skeleton fiber coil is not reported.

In this study, we present that the Faraday effect-induced bias error of PM-IFOG changes with temperature in the non-skeleton fiber coil. This phenomenon is caused by the temperature dependence of linear birefringence and Verdet constant of PM fiber. In particular, we verify that the Faraday effect-induced bias errors of two polarizations always have opposite signs that can be compensated optically regardless of the variation of the temperature.

## 2. Theory

**Figure 1:**
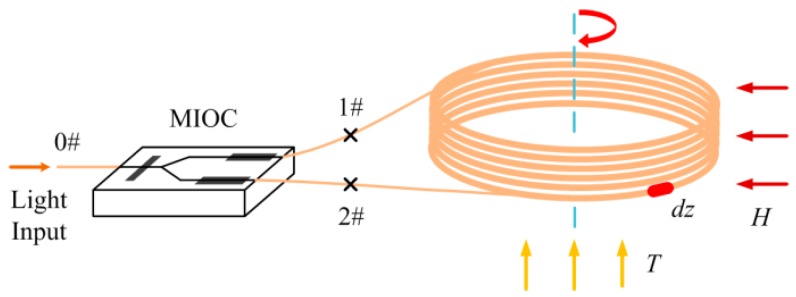
Portion of the IFOG optical circuit used in the analysis, including the MIOC and the PM fiber coil.

A typical IFOG configuration is shown in Figure 1, starting from the input/output port no. 0. The polarizer, beam splitter, and modulators are combined in a single multifunction integrated optic circuit (MIOC), which is butt-coupled to the PM fiber coil with *θ*_1_ and *θ*_2_ alignment angles at port no. 1 and no. 2. The coil is fabricated by Panda PM fiber with two polarization modes (denoted as *x* and *y*). When both *θ*_1_ and *θ*_2_ are 0°, the light beams are propagated in the *x* polarization mode. Alternatively, when both *θ*_1_ and *θ*_2_ are 90°, the light beams are propagated in the *y* polarization mode. The PM fiber coil, which is the main source of Faraday effect-induced bias error [18], is surrounded by the radial magnetic field *H* and temperature field *T.* The magnetic field is perpendicular to the sensitive axis of the PM fiber coil. The fiber with random twist is divided into micro-length unit *dz*. 

The Jones matrix method is conveniently used to calculate the bias error in IFOG [13]. In the presence of the radial magnetic field, the Jones matrix of clockwise (CW) light beam on segment *i* of the PM fiber coil is given by [26]

(1) $$U_{+}=\left[\begin{array}{cc} \cos\eta_{+}dz-j\frac{\Delta \beta }{2\eta_{+}}\sin\eta_{+}dz & -\frac{\xi_{i}+t_{i}}{\eta_{+}}\sin\eta_{+}dz\\ \frac{\xi_{i}+t_{i}}{\eta_{+}}\sin\eta_{+}dz & \cos\eta_{+}dz+j\frac{\Delta \beta }{2\eta_{+}}\sin\eta_{+}dz \end{array}\right],$$

where *η*_+_ = [(Δ*β*/2)^2^ + (*ξ_i_* + *t_i_*)^2^]^1/2^, Δ*β* is the linear birefringence of PM fiber, *ξ_i_* and *t_i_* are the circular birefringence of per unit length which is induced by Faraday effect and twist, respectively; and *ξ_i_* = *VH*sin(*dz*/*r*), *V* is the Verdet constant of the fiber, *r* is the radius of the PM fiber coil (*r* = 0.06 m). *t_i_* = (1 − *g*)*ϕ*, *ϕ* is the twist angle of per unit length, *g* is the photoelasticity coefficient of the fiber (about 0.06–0.08) [13].

Similarly, the Jones matrix of counter-clockwise (CCW) light beam on segment *i* of the PM fiber coil is given by

(2) $$U_{-}=\left[\begin{array}{cc} \cos\eta_{-}dz-j\frac{\Delta \beta }{2\eta_{-}}\sin\eta_{-}dz & -\frac{\xi_{i}-t_{i}}{\eta_{-}}\sin\eta_{-}dz\\ \frac{\xi_{i}-t_{i}}{\eta_{-}}\sin\eta_{-}dz & \cos\eta_{-}dz+j\frac{\Delta \beta }{2\eta_{-}}\sin\eta_{-}dz \end{array}\right],$$

where *η*_-_ = [(Δ*β*/2)^2^ + (*ξ_i_* − *t_i_*)^2^]^1/2^.

The Jones matrices of each segment for CW and CCW light beams are expressed by Equation (1) and Equation (2), respectively. Hence, the electric field components of the CW light beam from port no. 0 to port no. 0, *E*_+_, and that of the CCW light beam, *E*_-_, are derived as

(3) $$E_{+}=\left[\begin{array}{cc} 1 & 0\\ 0 & 0 \end{array}\right]R\left(\theta_{2}\right)\prod_{i=1}^{m}U_{+}R\left(-\theta_{1}\right)\left[\begin{array}{cc} 1 & 0\\ 0 & 0 \end{array}\right]E_{j}e^{-j\phi },$$

(4) $$E_{-}=\left[\begin{array}{cc} 1 & 0\\ 0 & 0 \end{array}\right]R\left(-\theta_{1}\right)\prod_{i=m}^{1}U_{-}R\left(\theta_{2}\right)\left[\begin{array}{cc} 1 & 0\\ 0 & 0 \end{array}\right]E_{j},$$

(5) $$R\left(\theta \right)=\left[\begin{array}{cc} \cos\theta & -\sin\theta \\ \sin\theta & \cos\theta \end{array}\right],(\theta =\theta_{1},\theta_{2}),$$

where *φ* = *φ_s_* + Δ*φ*(*t*), in which contains the Sagnac phase shift *φ_s_* and the modulation-induced phase Δ*φ*(*t*); *E_j_* = *E_x_* or *E_y_*, which are the light beam intensity of *x* and *y* polarization, respectively; and *m* is the number of the segments. 

It is noteworthy that the small off-diagonal components in *U*_+_ of Equation (1) and *U*_-_ of Equation (2) have no influence on the Faraday effect-induced bias error, regardless of the finite extinction ratio of the polarizer [13]. Therefore, the signals at the photo-detector (PD) are proportional to the corresponding light beam intensity, and the related Faraday effect-induced phase shifts are expressed as [16]

(6) $$I_{x}=2E_{x}^{2}(\phi +\phi_{x}),$$

(7) $$I_{y}=2E_{y}^{2}(\phi +\phi_{y}),$$

(8) $$\phi_{x}=\int_{0}^{L}\frac{4VH}{\Delta \beta }t(z)\sin(\frac{z}{r})dz,$$

(9) $$\phi_{y}=-\int_{0}^{L}\frac{4VH}{\Delta \beta }t(z)\sin(\frac{z}{r})dz,$$

where *I_x_* and *I_y_* are the signals at PD of *x* and *y* polarizations, respectively; *φ_x_* and *φ_y_* are the Faraday effect-induced phase shifts of *x* and *y* polarizations, respectively; and *L* is the length of the PM fiber coil (*L* = 1000 m).

The equivalent Faraday effect-induced bias error is obtained as

(10) $$\Omega_{x}=\int_{0}^{L}\frac{\lambda cVH}{\pi r\Delta \beta L}t(z)\sin(\frac{z}{r})dz,$$

(11) $$\Omega_{y}=-\int_{0}^{L}\frac{\lambda cVH}{\pi r\Delta \beta L}t(z)\sin(\frac{z}{r})dz,$$

where Ω*_x_* and Ω*_y_* are the Faraday effect-induced bias errors of *x* and *y* polarizations, respectively; *λ* is the light wavelength; and *c* is the velocity of light in a vacuum.

Apparently, Ω*_x_* and Ω*_y_* origin from the spatial frequency component of twist *t*(*z*) equal to the inverse of the turn, and they depend on the linear birefringence Δ*β* and the Verdet constant *V* of PM fiber [10]. During the process of drawing the fiber and winding the PM fiber coil, random twist is introduced inevitably. After the PM fiber coil is fabricated, the twist is a constant parameter. Consequently, when the temperature and radial magnetic field remain steady, the Faraday effect-induced bias errors of *x* and *y* polarizations are fixed and they have opposite polarities [24,25].

In conventional panda PM fiber, properties of core material and stress applied on core vary with temperature, and they lead to temperature dependence of the birefringence Δ*n* [27,28]. The linear birefringence Δ*β* = 2πΔ*n*/*λ*, and wavelength *λ* is 1550 nm, so Δ*β* also depends on temperature *T*. The relationship between Δ*β* and *T* can be expanded in a Taylor series around a temperature *T*_0_ as [29]

(12) $$\Delta \beta =\Delta \beta (T_{0})+\frac{d\Delta \beta }{dT}(T-T_{0}),$$

where Δ*β*(*T*_0_) = 2027 rad/m (Δ*n* = 5 × 10^−4^), *T*_0_ = 20 °C, and *d*Δ*β*/*dT* = −3.04 rad/m/ °C (Δ*n*/*dT* = −7.5 × 10^−7^/°C) for a panda PM fiber. 

Additionally, the inherent temperature dependence of Verdet constant is expressed as [30]

(13) $$V=V_{0}+\frac{dV}{dT}(T-T_{0}),$$

where *dV*/*dT* is 4.2 × 10^−8^ rad/m/mT/ °C, *V*_0_ = 6 × 10^−4^ rad/m/mT at 20 °C.

**Figure 2:**
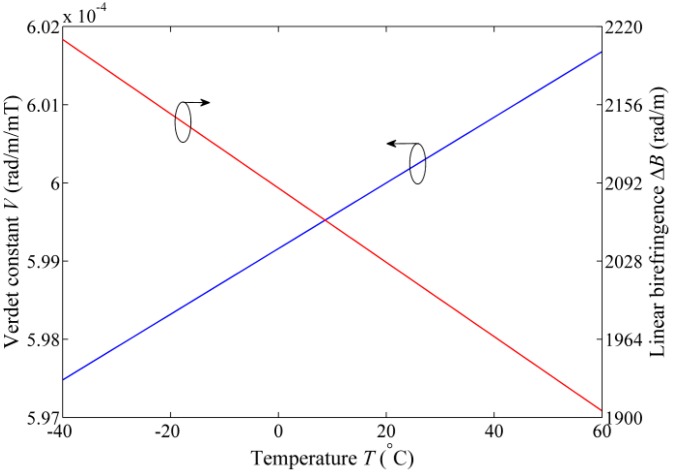
The temperature dependence of linear birefringence and Verdet constant.

Figure 2 presents the variation tendency of linear birefringence and Verdet constant due to temperature change for the panda PM fiber. Over the temperature range from −40 °C to 60 °C, the changes of the linear birefringence and Verdet constant are 304 rad/m and 4.2 × 10^−6^ rad/m/mT, respectively.

**Figure 3:**
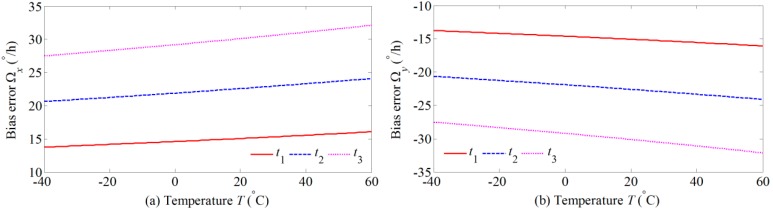
The temperature dependence of Faraday effect-induced bias errors: (**a**) *x* polarization; and (**b**) *y* polarization.

Substituting Equations (12) and (13) into Equations (10) and (11), the simulation results of the Faraday effect-induced bias errors of the *x* and *y* polarizations over the temperature range from −40 °C to 60 °C are plotted in Figure 3. Here, the radial magnetic field *H* is 1 mT, and the twists *t*_1_, *t*_2_, and *t*_3_ are 0.2sin(*z*/*r*), 0.3 sin(*z*/*r*), and 0.4sin(*z*/*r*), respectively. Obviously, the Faraday effect-induced bias errors of two polarizations change with temperature variation. Moreover, Faraday effect-induced bias errors of two polarizations always have opposite polarities even though the twist and temperature change.

**Figure 4:**
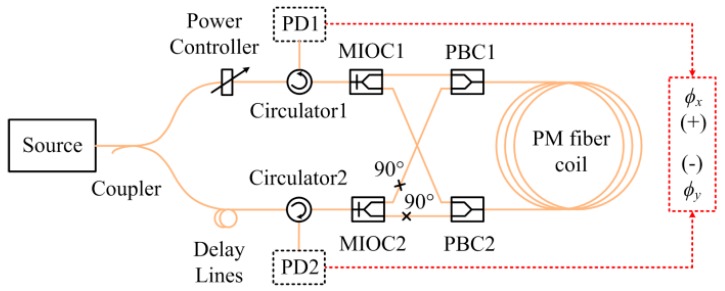
The configuration of dual-polarization IFOG, consisting of an amplified spontaneous emission (ASE) source (central wavelength 1550 nm, bandwidth 70 nm), a 3 dB single-mode coupler, two MIOCs, two polarization beam couplers (PBCs), two circulators and two PDs, a PM fiber coil.

As mentioned, the Faraday effect-induced bias error can be compensated optically at room temperature [24]. The optical compensation configuration is referred to the ‘dual-polarization IFOG’, in which two orthogonal polarizations propagate simultaneously in the PM fiber. The dual-polarization IFOG is illustrated in Figure 4. Light beam from ASE source is divided into two equally light beams by the coupler, and polarized by polarizers in the MIOCs. Because the fiber pigtails of MIOC2 are butt-coupled to two PBCs with the alignment angles of 90°, the light beams travel along two orthogonal directions in the PM fiber coil. When the light beams travel back, the orthogonal polarizations are separated by the polarizers and detected by the PDs. The power controller ensures that the light beam intensities are equal for two polarizations, and the incoherence of two polarizations is obtained by the optical delay lines. Therefore, two incoherent orthogonal polarization modes propagate simultaneously in the PM fiber coil with equal intensity.

When the signals at two PDs are summed up directly, the overall signal *I_sum_* and the Faraday effect-induced bias error Ω*_sum_* of dual-polarization IFOG are expressed as

(14) $$I_{sum}=2(E_{x}^{2}+E_{y}^{2})(\phi +\phi_{sum}),$$

(15) $$\Omega_{sum}=\frac{E_{x}^{2}\Omega_{x}+E_{y}^{2}\Omega_{y}}{E_{x}^{2}+E_{y}^{2}}.$$

Substituting Equations (10) and (11) into Equation (15), when the intensities of two polarizations are balanced (*E_x_* = *E_y_*), the Faraday effect-induced bias error can be eliminated by optical compensation regardless of the changes of the temperature. 

## 3. Experimental results

**Figure 5:**
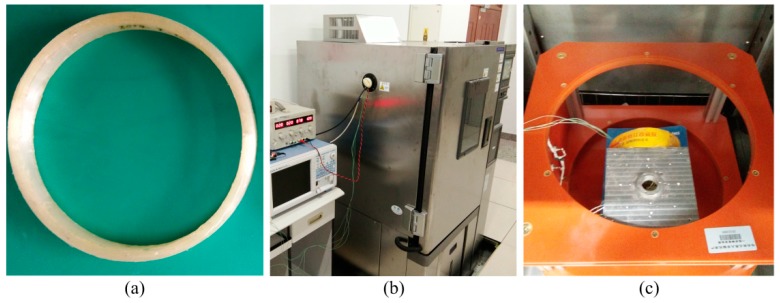
Experimental systems: (**a**) non-skeleton PM fiber coil; (**b**) temperature chamber; and (**c**) Helmholtz coil and PM fiber coil.

**Figure 6:**
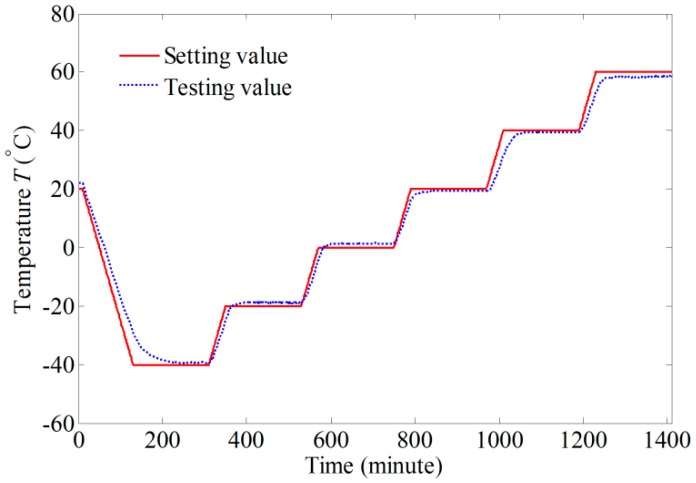
Setting and testing temperature.

To make the simulation results more persuasive, a dual-polarization IFOG is assembled according to the configuration shown in Figure 4. Figure 5 presents the experimental systems, which include a non-skeleton PM fiber coil (Harbin Engineering University), a temperature chamber (Espec environmental equipment company), and a Helmholtz coil (Harbin Engineering University). The parameters of the dual-polarization IFOG are the same as those in the simulation. The PM fiber coil is wound in a quadrupolar pattern to minimize the Shupe effect-induced error [31]. As shown in Figure 5c, the PM fiber coil is placed separately in the central region of the Helmholtz coil, where a nearly uniform magnetic field is produced. The sensitive axis of PM fiber coil is perpendicular to the magnetic field direction. Both the PM fiber coil and the Helmholtz coil are put into the temperature chamber. The temperature is controlled according to the red solid line shown in Figure 6. The temperature change rate is 0.5 °C/min, and the temperature holds for three hours at temperature points −40, −20, 0, 20, 40, and 60 °C, respectively. After the temperature of the PM fiber coil reaches these temperature points, the magnitude of the magnetic field is adjusted by altering current in the Helmholtz coil. The Faraday effect-induced bias error is the differences before and after applying the magnetic field. Two temperature sensors are mounted on the surfaces of the PM fiber coil and the measured temperatures can be approximately deemed as temperatures of the PM fiber coil. Although the measured temperatures of the PM fiber coil are different, the differences are minor and we only plot data acquired by one sensor as the blue dashed line in Figure 6. The output signals of IFOG are transmitted to a computer for storage and off-line processing.

**Figure 7:**
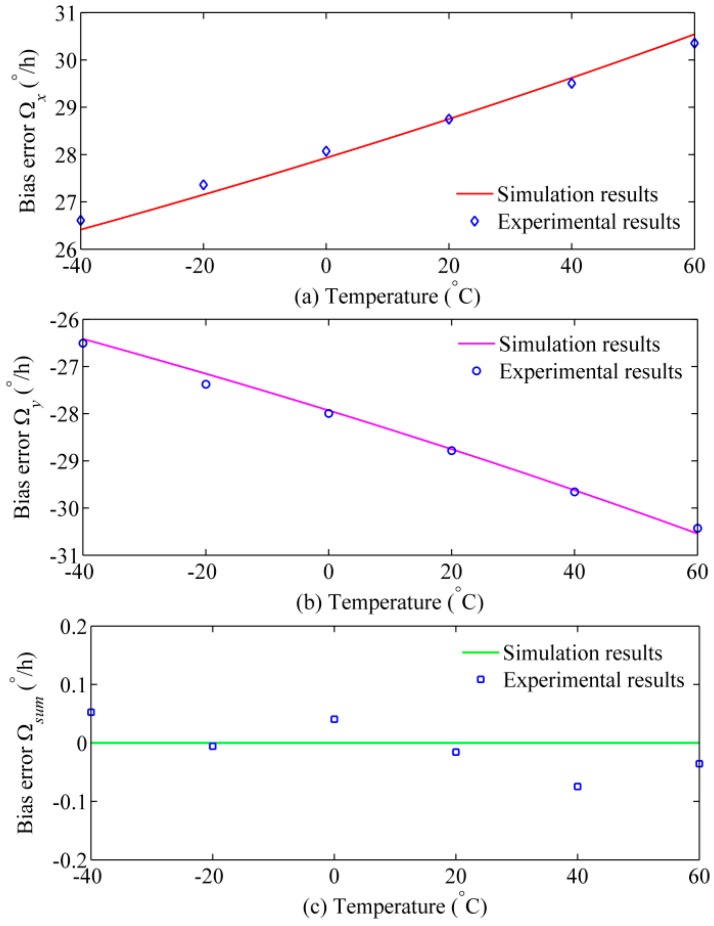
Simulation and experimental results of temperature dependence of Faraday effect-induced bias errors: (**a**) *x* polarization; (**b**) *y* polarization; and (**c**) optical compensation.

To eliminate the temperature dependence of the Helmholtz coil, the magnetic field intensity around the PM fiber coil is tested by two magnetic sensors with temperature compensation (QST Corporation, QMC5883L). Under the 1 mT radial magnetic field, the Faraday effect-induced bias errors at different temperature points are plotted as the blue points in Figure 7. As expected, the experimental results are completely consistent with theoretical predictions. The Faraday effect-induced bias error of *x* polarization changes from 26.61°/h to 30.36°/h when the temperature changes from −40 °C to 60 °C. Conversely, the Faraday effect-induced bias error of *y* polarization always has opposite signs when compared with that of *x* polarization. More importantly, although the temperature fluctuates dramatically, the compensated output appears a low magnetic sensitivity (about 0.08°/h/mT), which satisfies the precision requirement of navigation-grade IFOG. Fitting with the experimental data gives *t* = 0.382sin(*z*/*r*), Δ*n*/*dT* = −7 × 10^−7^/°C, and (*dV*/*dT*)*/V*_0_ = 0.7 × 10^−4^/°C, which are the same order as the experimental measurements in [27,30].

**Figure 8:**
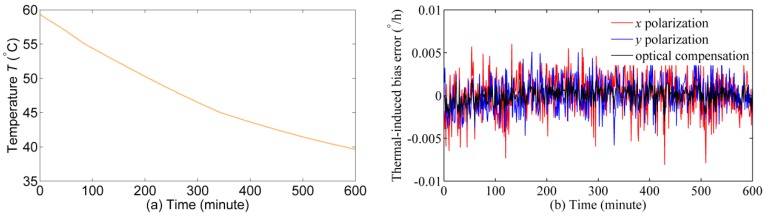
Experimental results: (**a**) temperature outputs; (**b**) thermal-induced bias error of *x* polarization, *y* polarization, and optical compensation.

To make the experiments more comprehensive, a comparison experiment is conducted to verify that the thermal-induced bias error has no influence on the previous experimental results. The PM fiber coil is placed in a *μ*-metal magnetic shield (the magnetic field intensity is reduced by 100 times) and then together put into the temperature chamber. The temperature of PM fiber coil is heated to about 60 °C at first, and then the temperature is dropped to about 40 °C naturally. The output signal of IFOG is processed by subtracting the mean value of the original signal. The experimental results are plotted in Figure 8.

## 4. Discussion

In Figure 7, the testing temperature of Faraday effect-induced bias errors is set at some fixed points (temperature change rate is approximate to 0 °C/min) for quantitative analysis and suppressing the Shupe effect-induced bias error [31]. Compared with the testing temperature of the Faraday effect-induced bias errors in Figure 7, the temperature in Figure 8a shows a larger fluctuation (temperature change rate range is −0.06 °C/min to −0.02 °C/min). Thus, the thermal-induced bias error in Figure 8b is larger than that in Figure 7. Figure 8b indicates that the thermal-induced bias error of *x* and *y* polarizations is lower than 0.01°/h when the temperature drop from 59.4 °C to 39.7 °C. Therefore, the thermal-induced bias error in Figure 7 should lower than 0.01°/h at the temperature range from 40 °C to 60 °C. In Figure 7, the changes of Faraday effect-induced bias errors of *x* and *y* polarizations are 0.77°/h when the temperature varies from 40 °C to 60 °C. As a result, the influence of thermal-induced bias error in the first experiment can be excluded.

As the theoretical model points out, the power balance is crucial for effective suppression of Faraday effect-induced bias error through optical compensation. In Figure 7c, there were still some small Faraday effect-induced bias errors after optical compensation, which may be caused by the imperfect balance of optical intensity in two polarizations. Generally, the IFOG on the market have a better performance on Faraday effect-induced bias error by shielding the fiber coil with multiple layers of magnetic shield. However, the Faraday effect-induced bias error in dual-polarization IFOG can be further suppressed by placing the fiber coil in a magnetic shield or using the air-core photonic bandgap fiber. If the fiber coil and the magnetic shield are the same, the Faraday effect-induced bias error after optical compensation will much lower than that of conventional IFOG. Additionally, the configuration of dual-polarization IFOG increases the structural complexity as more polarization selective elements are required. There are two reasons: observing the Faraday effect-induced bias errors of two polarizations simultaneously, and confirming the performance of optical compensation. However, the optical compensation configuration can be simplified, which has been demonstrated in [25,32].

It is noteworthy that the optical compensation mechanism allows the spatial temperature gradient along the fiber, which will induce the spatial dependence of birefringence. According to Equations (10) and (11), although the birefringence varies with fiber length, the Faraday effect-induced bias errors of two polarizations still show opposite signs that originate from the intrinsic symmetry of two orthogonal polarizations. Actually, the birefringence of the fiber changes along with fiber length due to the twist even at room temperature. In our previous work [24,25], the performance of the optical compensation for fiber coil with different twist is verified, which indicates the optical compensation allows spatial dependence of birefringence to some extent. 

All the theoretical and experimental results demonstrate that the Faraday effect-induced bias error changes with the temperature variation in the non-skeleton PM fiber coil. More importantly, the Faraday effect-induced bias errors of two polarizations always have opposite signs that can be compensated optically regardless of the changes of the temperature. Different from the thermal stress-induced birefringence changes with the variation of temperature in [26], the Faraday effect-induced bias error changes at different temperatures are caused by the intrinsic temperature dependence of birefringence and Verdet constant of PM fiber. Compared to that in the fiber coil with skeleton, the change of Faraday effect-induced bias error versus temperature in non-skeleton fiber coil is a little lower. This may be because the birefringence of fiber coil with skeleton is more sensitive to temperature variation. In addition, in our previous study [25], we have verified that the Faraday effect-induced bias error is suppressed in IFOG with double sensitivity in room temperature, which is also different from the focus of this paper.

## 5. Conclusions 

An investigation of Faraday effect-induced bias error of IFOG under varying temperature is presented. Theoretical analysis and experimental results indicate that the temperature dependence of the Faraday effect-induced bias error exists in the non-skeleton fiber coil, and it is caused by the change in the birefringence and the Verdet constant of PM fiber. Moreover, Faraday effect-induced bias errors of two polarizations always present opposite signs that can be compensated regardless of the changes of the temperature. This should be a promising feature to overcome the Faraday effect-induced bias error in the IFOG.
